# Novel Therapeutics in Alzheimer's Disease

**DOI:** 10.1155/2012/207969

**Published:** 2012-03-26

**Authors:** Marwan Sabbagh, Anton P. Porsteinsson, Anil Nair, Abdu Adem

**Affiliations:** ^1^Banner Sun Health Research Institute, 10515 W. Santa Fe Dr, Sun City, AZ 85351, USA; ^2^Alzheimer's Disease Care, Research and Education Program (AD-CARE) and Memory Disorders Clinic, University of Rochester School of Medicine and Dentistry, 435 East Henrietta Road, Rochester, NY 14620, USA; ^3^Quincy Medical Center and Alzheimer's Disease Center/Clinical Trials, 114 Whitwell St, Quincy, MA 02169, USA; ^4^Department of Pharmacology & Therapeutics, Faculty of Medicine & Health Sciences, UAE University, Al Ain, UAE

Alzheimer's disease is a progressive disorder characterized by significant cognitive, functional, and behavioral dysfunction. Progressive symptomatic decline appears inevitable at present even with the available therapies, and therefore additional treatment options are urgently needed for a growing epidemic that affects 27 million people worldwide. Currently there are 75 drugs in clinical trials and other 200 or more in development. The drugs being developed are targeting different intra- and extra-cellular targets as well as different mechanisms of action. They include both symptomatic and disease modifying approaches. Nevertheless, the development of drugs that: cross the blood brain barrier; exert an identifiable mechanism of action; have good safety and toxicity profiles; have a robust clinical benefit is 1 becoming increasingly daunting.

In this special edition of the International Journal of Alzheimer's Disease, we have three original submissions and five reviews accepted. The paper by A. J. Bishop et al. examines Centella asiatica extract in an AD mouse model. Centella asiatica (CA), common name gotu kola, is an Ayurvedic herb used to enhance memory and nerve function. Orally administered centella asiatica attenuated beta-amyloid-associated behavioral abnormalities in TG 2576 mice. Another original submission is by April and colleagues. In their study, they engineered specific SOFA-HDV ribozymes, a new generation of catalytic RNA tools, to decrease APP mRNA levels. Additionally, they demonstrated that APP-ribozymes are effective at decreasing APP mRNA and protein levels as well as A*β* levels in neuronal cells. Also there was an original submission by Ma et al. In their study, New Zealand rabbits were fed 2% cholesterol-enriched diet for 6 weeks as a model of BBB disruption in vivo and then were fed chow supplemented with 2% cholesterol with or without 5 mg/kg/d simvastatin for an additional 4 weeks to assess BBB integrity with and without simvastatin. They find that simvastatin improves disturbed BBB function both in vivo and in vitro.

In addition to the original submission, five reviews were accepted. The review by Ryan and colleagues provides a timely and critical review of immunotherapies being developed for the treatment of AD. This is of great interest because the clinical trials of passive immunotherapies are nearing conclusion of phase III studies. The review by Ladurner and colleagues reviews the literature on transcranial magnetic stimulation (TMS) for the treatment of AD. TMS is entering phase II studies in the US in 2012 as a symptomatic treatment for AD. Early data suggest a demonstrable benefit but long-term benefit is not established. The review by Beccano-Kelly summarizes the body of knowledge around leptin as a potential treatment for AD since dysfunctions in the leptin system have recently been linked to neurodegenerative disorders such as Alzheimer's disease. This is based on the observation that leptin has widespread action in the CNS, and evidence is growing that leptin has the capacity to modulate higher brain functions. Leptin appears to have an effect on hippocampal-dependent function and in particular learning and memory processes. The review by Mehta et al. reviews new and developing acetylcholinesterase inhibitors as symptomatic treatments for AD. It turns out that there are dozens of synthetic analogues and hybrid compounds far beyond the approved donepezil, tacrine, rivastigmine, and galantamine. The review by Berk and Sabbagh that looks critically at the justification of using the new higher doses of donepezil in the treatment of moderate to severe Alzheimer's disease.

*Marwan Sabbagh* *Marwan Sabbagh* *Anton Porsteinsson* *Anton Porsteinsson* *Anil Nair* *Anil Nair* *Abdu Adem* *Abdu Adem*

